# Supplementary material for: Indirect regulation of HMGB1 release by gasdermin D
Source: Nat Commun. 2020 Sep 11;11:4561. doi: 10.1038/s41467-020-18443-3 (PMC7486936; doi:10.1038/s41467-020-18443-3)
Supplement: Supplementary file 1 — Supplementary Information [file 41467_2020_18443_MOESM1_ESM.pdf]

Supplementary Figure 1

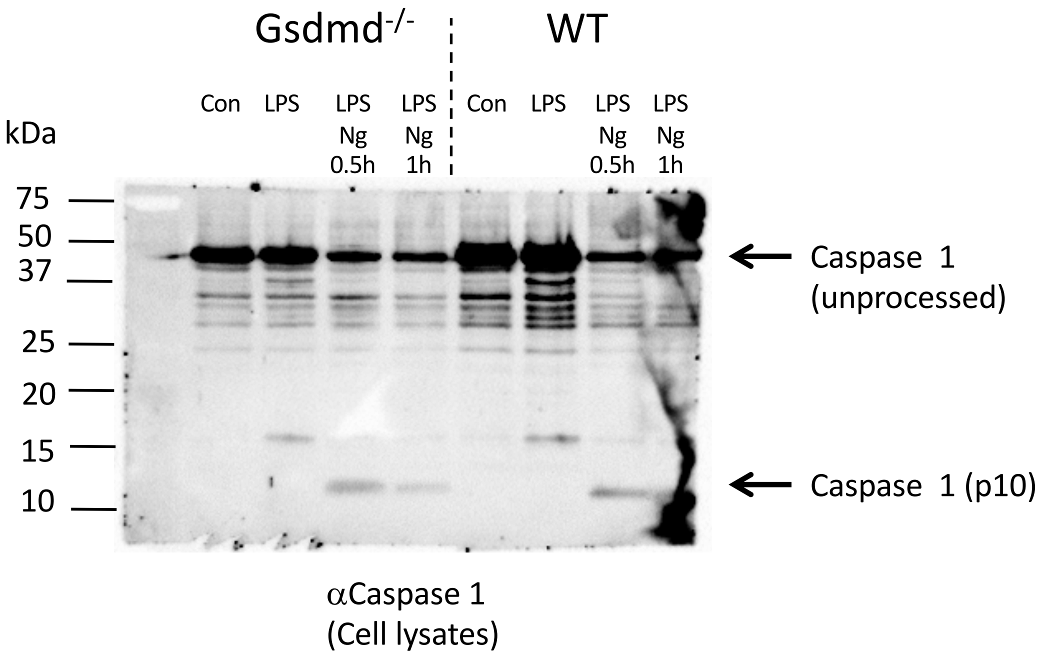

Supplementary Figure 1 – Pyroptosis-induced caspase 1 processing in BMDM. BMDM were prepared from wild type (WT) or gasdermin D knockout (*$Gsdmd^{-/-}$*) mice. The cells were primed or not with LPS (0.5 μg ml^{-1}) for 5h and treated with or without nigericin (Ng, 20 μM) as indicated. Cell lysates were prepared and immunobloted with an anti-caspase 1 antibody. Note the appearance of the p10 fragment following LPS + Ng treatment, indicative of inflammasome activation. Data are representative of results from n=3 independent experiments.
