# Supplementary material for: Indirect regulation of HMGB1 release by gasdermin D
Source: Nat Commun. 2020 Sep 11;11:4561. doi: 10.1038/s41467-020-18443-3 (PMC7486936; doi:10.1038/s41467-020-18443-3)
Supplement: Supplementary file 3 — Reporting Summary [file 41467_2020_18443_MOESM3_ESM.pdf]

nature research

Corresponding author(s): Benjamin E. Steinberg, Neil M. Goldenberg

Last updated by author(s): Aug 5, 2020

# Reporting Summary

Nature Research wishes to improve the reproducibility of the work that we publish. This form provides structure for consistency and transparency in reporting. For further information on Nature Research policies, see our Editorial Policies and the Editorial Policy Checklist.

## Statistics

For all statistical analyses, confirm that the following items are present in the figure legend, table legend, main text, or Methods section.

| n/a | Confirmed | |
|---|---|---|
| ☐ | ☒ | The exact sample size (*n*) for each experimental group/condition, given as a discrete number and unit of measurement |
| ☐ | ☒ | A statement on whether measurements were taken from distinct samples or whether the same sample was measured repeatedly |
| ☐ | ☒ | The statistical test(s) used AND whether they are one- or two-sided<br>*Only common tests should be described solely by name; describe more complex techniques in the Methods section.* |
| ☒ | ☐ | A description of all covariates tested |
| ☒ | ☐ | A description of any assumptions or corrections, such as tests of normality and adjustment for multiple comparisons |
| ☐ | ☒ | A full description of the statistical parameters including central tendency (e.g. means) or other basic estimates (e.g. regression coefficient) AND variation (e.g. standard deviation) or associated estimates of uncertainty (e.g. confidence intervals) |
| ☐ | ☒ | For null hypothesis testing, the test statistic (e.g. *F*, *t*, *r*) with confidence intervals, effect sizes, degrees of freedom and *P* value noted<br>*Give P values as exact values whenever suitable.* |
| ☒ | ☐ | For Bayesian analysis, information on the choice of priors and Markov chain Monte Carlo settings |
| ☒ | ☐ | For hierarchical and complex designs, identification of the appropriate level for tests and full reporting of outcomes |
| ☒ | ☐ | Estimates of effect sizes (e.g. Cohen's *d*, Pearson's *r*), indicating how they were calculated |

*Our web collection on statistics for biologists contains articles on many of the points above.*

## Software and code

Policy information about availability of computer code

| | |
|---|---|
| Data collection | No software was used for data collection. |
| Data analysis | GraphPad Prism Version 7.0; Volocity Version 6.5 |

For manuscripts utilizing custom algorithms or software that are central to the research but not yet described in published literature, software must be made available to editors and reviewers. We strongly encourage code deposition in a community repository (e.g. GitHub). See the Nature Research guidelines for submitting code & software for further information.

## Data

Policy information about availability of data

All manuscripts must include a data availability statement. This statement should provide the following information, where applicable:

- Accession codes, unique identifiers, or web links for publicly available datasets
- A list of figures that have associated raw data
- A description of any restrictions on data availability

All data generated during this study are included in this published article (and its supplementary information files).

# Field-specific reporting

# Life sciences study design

All studies must disclose on these points even when the disclosure is negative.

| | |
|---|---|
| Sample size | Sample sizes were assigned based upon historical practices, in line with recent relevant publications, for instance Evavold et al.The Pore-Forming Protein Gasdermin D Regulates Interleukin-1 Secretion from Living Macrophages. Immunity 48:35-44 (2018), as well as Ruhl et al. ESCRT-dependent membrane repair negatively regulates pyroptosis downstream of GSDMD activation. Science 362:956-960 (2018). |
| Data exclusions | No data were excluded from analysis. |
| Replication | All experiments were replicated independently at least 3 times, as indicated in figure legends. |
| Randomization | Cell samples were randomly allocated to treatment by random division into multi-well plates. Animals were randomized by random number. |
| Blinding | Investigators were blinded to group allocation during data collection and analysis for both in vitro and in vivo experiments. |

# Reporting for specific materials, systems and methods

We require information from authors about some types of materials, experimental systems and methods used in many studies. Here, indicate whether each material, system or method listed is relevant to your study. If you are not sure if a list item applies to your research, read the appropriate section before selecting a response.

## Materials & experimental systems

| n/a | Involved in the study | |
|---|---|---|
| ☐ | ☒ | Antibodies |
| ☒ | ☐ | Eukaryotic cell lines |
| ☒ | ☐ | Palaeontology and archaeology |
| ☐ | ☒ | Animals and other organisms |
| ☒ | ☐ | Human research participants |
| ☒ | ☐ | Clinical data |
| ☒ | ☐ | Dual use research of concern |

## Methods

| n/a | Involved in the study | |
|---|---|---|
| ☒ | ☐ | ChIP-seq |
| ☒ | ☐ | Flow cytometry |
| ☒ | ☐ | MRI-based neuroimaging |

## Antibodies

| | |
|---|---|
| Antibodies used | HMGB1 (Abcam, ab79823); IL-1b (R&D Systems, AF-401-NA); pro-caspase-1+p10+p12 (Abcam, ab179515); NLRP3 (AdipoGen, AG-208-0014); Gsdmd (Santa Cruz, sc-393656); GAPDH (Santa Cruz, sc-25778); ASC (BioLegend, #653902); AF488-Goat anti-Rabbit (ThermoFisher A-1108) |
| Validation | As per each respective manufacturer:<br>HMGB1 - knockout validated (Abcam)<br>Il-1b - validated for detection of mouse IL-1B, less than 15% cross reactivity with porcine (R&D systems)<br>pro-caspase-1 - recombinant antibody suitable for detection of mouse caspase-1 (Abcam)<br>NLRP3 - recognizes mouse and human NLRP3/NALP3 (AdipoGen)<br>Gsdmd - specific for an epitope mapping between amino acids 169-188 within an internal region of GSDMDC1 of mouse origin (Santa Cruz)<br>GAPDH - recognizes human and mouse GAPDH (Santa Cruz)<br>ASC - reacts with mouse, full length recombinant PYCARD (NP_037390) produced in E. coli (BioLegend)<br>AF488 Goat Anti-mouse - highly specific gor Rabbit IgG heavy and Light chains (ThermoFisher) |

## Animals and other organisms

Policy information about studies involving animals; ARRIVE guidelines recommended for reporting animal research

| | |
|---|---|
| Laboratory animals | Mice (Mus musculus) on a C57Bl/6 background, between 8-10 weeks of age, from mixed-sex cohorts were used. Animals were housed with free access to food and water, with a light:dark cycle of 14:10 hours. Ambient temperature is 22-24 degrees Celsius, and 45-55% relative humidity. |
| Wild animals | Study did not involve wild animals |
| Field-collected samples | Study did not involve field-collected samples |
| Ethics oversight | All animal procedures were approved by the Animal Care Committee at the Hospital for Sick Children and were performed in accordance with regulations and standards from the Animals for Research Act of Ontario and the Canadian Council of Animal Care |

Note that full information on the approval of the study protocol must also be provided in the manuscript.
